# The green healer: an updated review on the phytochemical profile and therapeutic potential of *Aloe vera*

**DOI:** 10.3389/fnut.2025.1689700

**Published:** 2025-09-30

**Authors:** Aimen Abid, Mavra Javed, Saira Zafar, Syeda Andleeb Zahra Hamdani, Syed Hassan Bin Usman Shah, Juweria Abid, Abdul Momin Rizwan Ahmad

**Affiliations:** ^1^Department of Nutrition & Dietetics, National University of Medical Sciences (NUMS), Rawalpindi, Pakistan; ^2^Department of Food Science and Human Nutrition, Michigan State University, East Lansing, MI, United States; ^3^School of Public Health, Health Services Academy, Islamabad, Pakistan; ^4^Armed Forces Postgraduate Medical Institute (AFPGMI), National University of Medical Sciences (NUMS), Rawalpindi, Pakistan; ^5^The Kirby Institute, University of New South Wales, Sydney, NSW, Australia; ^6^Department of Human Nutrition and Dietetics, NUST School of Health Sciences, National University of Sciences & Technology (NUST), Sector H-12, Islamabad, Pakistan; ^7^Department of Health Sciences, University of York, York, United Kingdom

**Keywords:** *aloe vera*, anti-inflammatory effects, antimicrobial activity, antioxidant properties, bioactive compounds, therapeutic potential

## Abstract

*Aloe vera* has historically been recognized as a versatile medicinal plant, attributable to its extensive array of therapeutic properties. Recent scientific research has shown a diverse variety of bioactive compounds in *Aloe vera* that contribute to its potential pharmacological capabilities. This review synthesizes contemporary data on the phytochemistry, health benefits, and potential clinical applications of *Aloe vera*. Anthraquinones, flavonoids, polysaccharides, and other bioactive substances found in *Aloe vera* exhibit significant anti-inflammatory, antioxidant, antibacterial, and immunomodulatory effects. Furthermore, its nutritional composition is exceptionally varied, since *Aloe vera* contains prominent vitamins, minerals, and amino acids, rendering it an outstanding supplement for sustaining overall health. Its applications have been validated in the therapy of metabolic disorders, gastrointestinal recovery, improvement of dermatological conditions, and wound healing. *Aloe vera* continues to garner interest in both conventional and contemporary healthcare systems due to its affordability, accessibility, and safety profile as an integrative approach.

## 1 Introduction

*Aloe vera* is a genus of succulent plants renowned for its exceptional therapeutic and medical properties, therefore gaining global recognition and appreciation across numerous civilizations throughout history (1). Complex carbohydrates of aloe are plentiful sources of natural complex carbohydrates, including those derived from plants (e.g., pectin, guar gum), animals (e.g., chitosan), and microbes (e.g., dextran). Specific plant-derived carbohydrates, such as those present in aloe, are acknowledged for their diverse biological activities. Aloe, formerly classified in the Liliaceae family, has been reclassified into the Aloaceae family. The plant, native to South and East Africa and the Mediterranean region, consists of approximately 400 species that are now cultivated worldwide, particularly thriving in subtropical environments (2). *Aloe vera* is a robust, evergreen, shrubby succulent characterized by green, thick leaves that exhibit hues ranging from green to gray, arranged in a rosette formation around a central stem. The leaves have serrated triangular shapes and consist of a robust outer layer (epidermis) with a cuticular layer that encases the water-retaining tissue within the mesophyll. This distinctive configuration enables the plant to conserve water well, hence thriving in arid conditions and prolonged droughts. The plant produces tubular flowers that may be solitary or clustered, with coloration varying by type (3).

Among Aloe species, vera is the most extensively researched and possesses the highest commercial value due to its potent pharmacological properties. Historically, its therapeutic use extends to ancient civilizations (4). The ancient Egyptians referred to it as the plant of immortality, vital to embalming practices. Meanwhile, Greeks and Romans utilized it for its therapeutic effects in treating inflammations and skin blemishes. The longstanding traditional use has prompted contemporary scientific investigation into its active components (5). *Aloe vera* possesses a unique amalgamation of nutrients, encompassing various vitamins, minerals, amino acids, and enzymes, which enhances its therapeutic efficacy in addressing numerous health conditions, particularly those affecting the skin and digestive system (6). The medicinal properties of Aloe leaf extracts are mostly linked to the presence of polysaccharides found in the interior parenchymatous tissue of the leaf (7). It is, however, acknowledged that the health advantages of the plant arise from the synergistic effects of its several components, rather than the influence of a singular molecule. The therapeutic properties of *Aloe vera* are mostly associated with its phytochemical composition. The most significant bioactive constituents encompass compounds recognized for their potent anti-inflammatory, antibacterial, and antioxidant properties, namely anthraquinones, polysaccharides, and glycoproteins (8). These attributes establish *Aloe vera* as a pragmatic and efficacious herbal remedy in both traditional and contemporary medicinal contexts. Recent studies have demonstrated that *Aloe vera* gel considerably enhances wound healing and reduces inflammation, hence reinforcing the plant’s reputation as a preferred treatment for burns, cuts, and skin injuries (9). Moreover, modern science validates the efficacy of *Aloe vera* beyond mere topical treatment. A study demonstrated that *Aloe vera* positively influences blood parameters in rats and may be regarded as a natural medicinal resource (10).

Furthermore, research on *Aloe vera* juice has revealed beneficial health benefits on inside human physiology, perhaps assisting in digesting, reducing symptoms of irritable bowel syndrome, and enhancing immunological response. *Aloe vera*’s adaptability and efficacy in skincare render it a natural gift, particularly with the advancement of polyherbal formulations that include *Aloe vera*, recognized as some of the most efficient skin creams (11).

The therapeutic potential of *Aloe vera* is continually investigated, underscoring its significance in both ancient and contemporary medicine. Researchers continue to explore the extensive array of bioactive chemicals it possesses, indicating significant potential for identifying novel and enhanced applications for this extraordinary plant. The increasing scientific proof of its health benefits highlights the significance of *Aloe vera* in complementary medicine and stimulates deeper exploration of its mechanisms of action. This summary seeks to encapsulate existing knowledge regarding the bioactive components of *Aloe vera* and their health-enhancing benefits, underscoring the necessity for ongoing study to fully exploit its medicinal potential. *Aloe vera* is the most extensively commercialized among the over 400 kinds of aloe. The extraction of its leaf pulp has evolved into a worldwide industry, with the pharmaceutical sector employing it in the manufacture of topical formulations (including ointments and gels) as well as tablets and capsules (12).

## 2 Nutritional profile of *Aloe vera*

Fresh *Aloe vera* leaves consist of water (90–99%), with the remainder being carbohydrates, proteins, and minerals. The dry matter comprises 50–70% carbs, 10–15% proteins, and around 7% ash. The plant contains numerous amino acids, several of which are essential for human health, in addition to vitamins A, C, E, B-complex, and folate (B9). *Aloe vera* contains essential minerals such as calcium, magnesium, and phosphorus, along with trace elements including zinc, selenium, copper, and chromium (13). These nutrients contribute to the plant’s overall dietary value; nevertheless, the quantities typically consumed by individuals are insufficient to significantly impact their nutrition. The nutrient composition varies significantly depending on the plant portion (gel versus leaf powder), cultivation methods, and processing techniques, complicating direct comparisons of research findings. *Aloe vera* contains substantial quantities of macronutrients and micronutrients; nevertheless, its primary significance in human health research is in its bioactive phytochemicals rather than as a source of essential nutrients (14).

### 2.1 Carbohydrates

Complex carbohydrates make *Aloe vera* rich in mono- and polysaccharides, which form the plant’s interior gel encased in a protective coating. Using a polyuronide with a glucose-mannose molecular weight, the gel’s carbohydrate composition was mannose, glucose, and uronic acid. Other sugars in the gel include galactose, arabinose, and xylose. *Aloe vera* gel’s saponins are cleaning and antibacterial, which is good. Due to its unique chemistry and bioactive components, the plant is a general-purpose herbal treatment with several medical purposes (15). *Aloe vera* gel and latex fractions have been utilized therapeutically since ancient times. Since plant cells use the yellow latex and clear gel most, they are used as liquid portions. Health professionals employ them (16). They are galectin, pectic material, galactogalacturan, xlan, pure mannan, acetylated mannan, glucomannan and arabinogalactan. The gel material is made up of aloe carbohydrates which are the ones behind the plant soothing and moistening effects which are very important in soothing burns and skin irritations (17). Polysaccharides, such as glucomannan enhance the activity of fibroblasts and collagen synthesis which enhances wound healing. The immunomodulatory properties of theirs are also helpful in providing the enhancement of defensive power of the body by the activation of the macrophages and other immuno-competent cells (18).

### 2.2 Proteins and amino acids

*Aloe vera* has a minimum of 20 amino acids. The amino acids comprise Arginine, histidine, hydroxyproline, aspartic acid, glutamic acid, proline, glycine, tyrosine, alanine, isoleucine, leucine, lysine, threonine, valine, phenylalanine, as well as lectins and lectin-like substances. Arginine is relatively abundant, constituting around 20% of the total amino acids (19).

### 2.3 Vitamin and minerals

*Aloe vera* is rich in vital vitamins including vitamins A, B complex, C, E and D. Furthermore, it is proposed by some studies that the plant can have a small amount of vitamin B12. Such enzymes as carboxypeptidase possessing anti-inflammatory properties and other proteins, including amylase, lactic dehydrogenase, and lipase, have also been identified in the gel of *Aloe vera* (20). The plant further contains minerals such as sodium, potassium, calcium and magnesium at varying proportions or found in different parts of the plant. It is worth noting that the main mineral content of the skin is calcium whereas sodium and potassium are more than abundantly present in the gel. There are still other minerals, such as aluminum, iron, and chloride which have been identified in *Aloe vera* gel (21). The properties of *Aloe vera* in terms of merging the set of vitamins, the presence of enzymes, and minerals also become a source of its possible health benefits and therapeutic functions. More studies of the nutritional content of *Aloe vera* can open new valuable horizons in the application of this plant in folk medicine and contemporary medical practice (22).

### 2.4 Moisture

The interior gel of *Aloe vera* predominantly consists of water, with moisture content estimates ranging from 98 to 99.5 percent. The exceptionally high-water content is a primary factor enabling *Aloe vera* to provide profound moisturization, soothing, and hydrating properties on the skin. The substantial water content in the gel serves as an exceptional natural remedy for dry, irritated, or inflamed skin, providing prolonged hydration and soothing effects (23).

### 2.5 Fiber

*Aloe vera* is abundant in fiber, a type of complex carbohydrate, in addition to its moisture content. *Aloe vera* can occasionally exhibit elevated levels of crude fiber due to differences in its source or processing; akin to other plants, this crude fiber is found in the leaf pulp and may be extracted as stem fibers. Research studies have explored the potential applications of *Aloe vera* fiber, including the production of composite materials (24). According to published studies in the ARCC Journals, the fiber content in the leaf pulp of *Aloe vera* is approximately 16.8% (25). The fiber content enhances the plant’s utility beyond its moisturizing properties, demonstrating its adaptability and the potential for additional research and development.

## 3 Phytochemical profile of *Aloe vera*

Research on *Aloe vera*’s phytochemicals has shown a rich array of bioactive compounds. The total flavonoid and phenol content of *Aloe vera* extract was examined using HPLC to determine its therapeutic potential. Hydroxycinnamic acids, arthrons, and chromones were abundant in leaf rind (26). Phenolic chemicals vary by plant component, especially between leaf epidermis and flowers. Catechin and gentisic acid dominate these tissues. *Aloe vera* flower ethanol extracts have been evaluated for phenolic and flavonoid content (26). The allocation of these beneficial molecules differs among plant tissues: the rind is often more abundant in phenolic compounds, whereas flowers exhibit unique patterns of fatty acids and flavonoids (27). The variability in phytochemical composition is associated with parameters like extraction process, plant age, and environmental circumstances, highlighting the necessity for consistent methodologies in phytochemical characterization (27).

### 3.1 Antioxidants

The interest in studying the pharmacological and phytochemical potential of the plant arises from its antioxidative properties and its potential utility in treating gastrointestinal diseases, stimulating skin tissue regeneration, and exhibiting anti-inflammatory and antioxidant effects, as corroborated by research (28). The bioactive components in *Aloe vera* can positively influence the reduction of lipid and polysaccharide metabolism, cholesterol levels, and the stabilization of blood glucose (13). Recent research have identified the anti-cancer properties of the plant due to the presence of chemicals such as aloe-emodin and anthraquinone (29). *Aloe vera* is generally regarded as a promising subject for further investigation into its health benefits and potential therapeutic applications.

### 3.2 Phenolic compounds

*Aloe vera* contains a diverse array of phytochemicals, which may contribute to its potential health benefits and therapeutic characteristics. Polyphenolics, flavonoids, tannins, and anthraquinones are associated with various functional activities, including antioxidant, anti-inflammatory, and antibacterial properties.

These compounds can mitigate oxidative stress-related diseases and provide anti-infective properties. The phytochemical composition of *Aloe vera* includes a variety of bioactive chemicals, such as aloesin, catechin, and genistic acid, which are present in different regions of the plant in varying amounts (91). The phenolic content of *Aloe vera* leaf skin is significantly more than that of its flowers. The potential phytochemical health benefits of *Aloe vera* provide this plant with a promising subject for further exploration of its therapeutic applications. Understanding the quantitative and qualitative characteristics of the phytochemicals in *Aloe vera* is essential for harnessing its medicinal potential and exploring its use in both modern and traditional medicine (30).

### 3.3 Anthraquinones/anthrones

*Aloe vera* contains many anthraquinones and Anthrones derivatives, including Aloe-emodin, aloetics, anthranol, barbaloin, cinnamic acid esters, chrysophanic acid, anthracene, babendil, microdontic, as well as glycosylated and methylated derivatives. The anthraquinones, derivatives of the quinone family, are crucial to the therapeutic properties of Aloe (31). These chemicals are recognized as laxatives; nevertheless, newer research has discovered additional effects. Anthraquinones exhibit antioxidative, antiviral, and cytotoxic properties, particularly against squamous cell lung carcinoma and Streptococcus viridians. Research is being conducted to explore their potential application in the treatment of malaria, viral diseases, and fungal infections. Aloe contains two primary anthraquinones: aloe-emodin and aloin. Aloe juice is rich in aloe-emodin, which is available in powdered form as a laxative (32). Anthraquinones undergo oxidation/reduction in the colon, converting to anthranol and Anthrones, which increase intestinal secretion, peristalsis, and limit water absorption. Aloe-emodin exhibits remarkable reducing capacity and hydroxyl free radical scavenging ability, consequently inhibiting the oxidation of linolenic acid by 78%. It exhibits cytotoxicity against squamous cell lung cancer cells by inducing apoptosis through the activation of caspase enzymes. Aloe-emodin: an Aloe-emodin Anthrones C-glucoside and aloin are similar in this respect, primarily functioning as laxatives. It additionally inhibits lipid peroxidation in the cerebral cortex by inactivating Fe (II)-dependent ascorbate, indicating its use beyond its laxative properties (33).

### 3.4 Flavonoids

*Aloe vera* contains these compounds: genistein, is vitexin, naringenin, apigenin, and dihydro isorhamnetin. Plants include naturally occurring polyphenolic chemicals that can reduce inflammation, protect against cancer, and oxidative stress. In addition to supporting cardiovascular health and the immune system, they play an important function in skin health by neutralizing free radicals and reducing oxidative damage on cells (34) Additionally, the flavonoids in *Aloe vera* help explain why this plant helps reduce inflammation in skin illnesses, blood circulation in capillaries, and histamine release. To create a synergistic impact in healing and reducing oxidative stress, they can enhance the use of various *Aloe vera* products (17).

### 3.5 Chromones

Numerous chromones identified in *Aloe vera* include 8-C glucosyl, 7-O-methyl-aloediol, isoaloeresine D, isorabaichromone, and neoaloesin A. The primary benefits of Aloe’s anti-inflammatory and anti-allergic properties are realized through these chromone derivatives. Other agents have been identified to inhibit the production of histamine and leukotrienes, therefore regulating allergic responses and inflammation (35). Furthermore, chromones are exclusive to Aloe species and are quite rare in other plants, making them effective phytochemical markers. Their antibacterial and analgesic properties account for the plant’s traditional usage in treating wounds and digestive issues (36).

### 3.6 Glycoproteins

*Aloe vera* includes unique lectins known as aloctin A and B. The exhibited lectins demonstrate distinct characteristics regarding the interactions between the oligosaccharide group and the polypeptide chain. Aloctin A is glycosylated via serine or threonine, whereas aloctin B employs asparagine for glycosylation. Lectins have a crucial role in facilitating cell division and augmenting the proliferation of B- and T- lymphocytes, hence exhibiting mitogenic and immunochemical properties. They facilitate cellular blastic transformation, transitioning the cell from the resting phase to the synthesis phase or interphase, ultimately leading to mitotic divisions. Aloctin possess the ability to agglutinate and eliminate malignant cells by adhering to the polysaccharide fragments found in the membranes of these cells (37).

## 4 Therapeutic potential of *Aloe vera*

**Figure 1:**
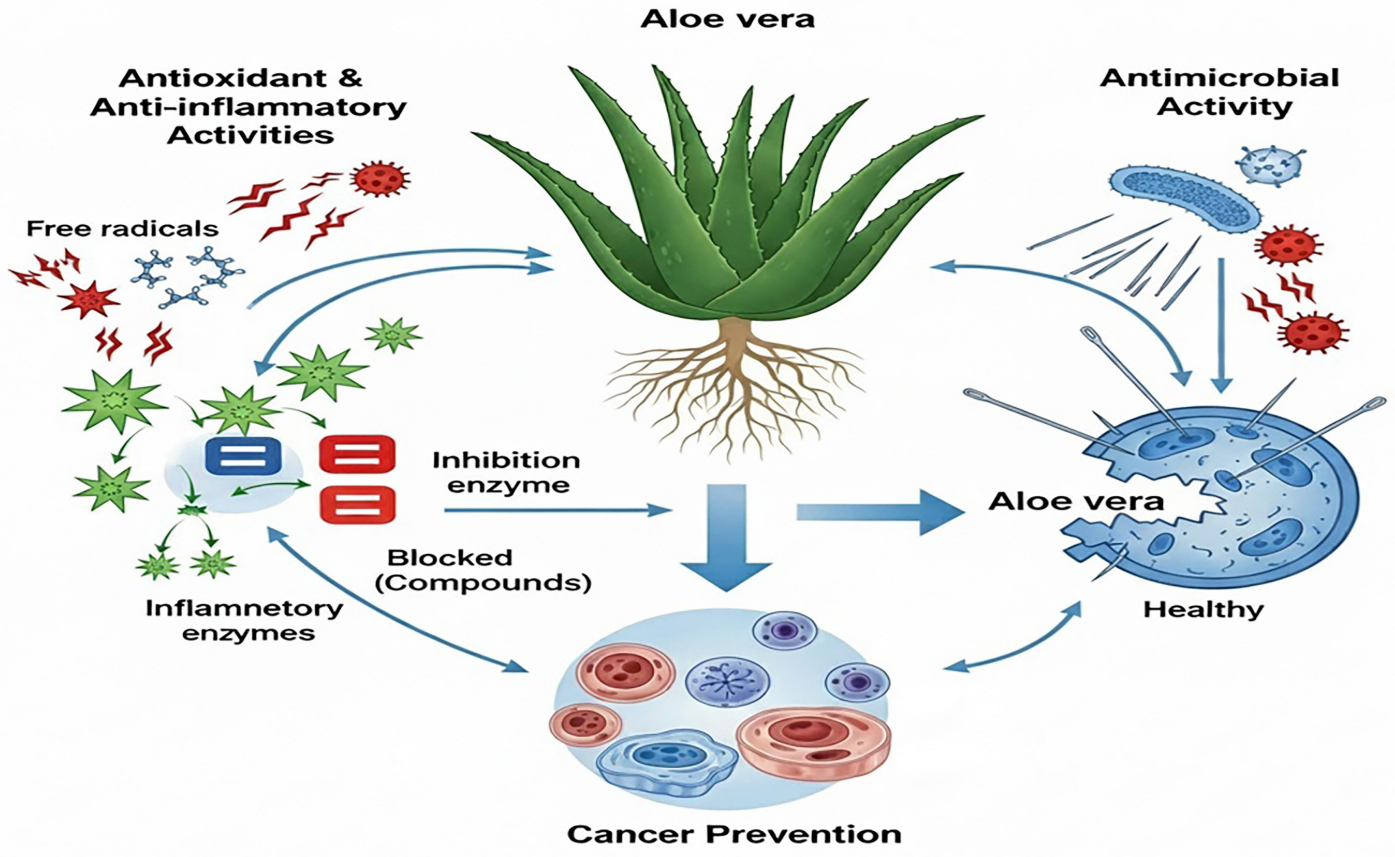
Possible mechanism of action of *Aloe vera.*

*Aloe vera* has become prominent in the natural medicine industry due to its potential health advantages. Researchers assert that this multifaceted plant may significantly aid various health-related therapies. Research demonstrated the hepatoprotective effects of *Aloe vera* extract in male Wistar albino rats, evidenced by a significant reduction in serum alanine transaminase levels, suggesting that its consumption may ensure liver safety alongside additional hematological benefits (38). Furthermore, the nutritional richness of *Aloe vera* underscores its role in addressing dietary imbalances and providing a wealth of essential elements that can enhance general health. These findings underscore the diverse therapeutic potential of the plant, hence supporting its application in both preventive and remedial health interventions. Consequently, recent study suggests that *Aloe vera* may serve not only as a treatment for exterior issues but also as a crucial element in enhancing overall health (39). Table 1 and Figure 1 delineate the therapeutic potential of *Aloe vera*, the associated bioactive chemicals, and their mechanisms of action.

**Table 1:** Therapeutic potential of *Aloe vera*, bioactive compounds and mechanism of action.

Therapeutic potential	Bioactive compounds involved	Mechanism of action	References
Antioxidant activity	Acemannan, Aloe-emodin	Scavenging free radicals, reducing oxidative stress	(71)
Anti-inflammatory effects	Aloin, Acemannan	Inhibition of pro-inflammatory cytokines	(72)
Wound healing	Acemannan, *Aloe vera* gel	Promotes fibroblast proliferation and collagen synthesis	(73)
Antimicrobial activity	Aloe-emodin, Anthraquinones	Disruption of microbial cell membranes	(74)
Anticancer effects	Aloin, Aloe-emodin	Induction of apoptosis and cell cycle arrest	(75)
Skin hydration	Acemannan	Enhance moisture retention in skin	(18)
Antidiabetic effects	Acemannan	Modulation of glucose metabolism	(76)
Gastroprotective effects	Aloin	Mucosal protective effects against ulcers	(77)
Antiviral activity	Acemannan	Inhibition of viral replication	(78)
Immune system modulation	Acemannan	Stimulation of immune cell activity	(79)
Hair growth promotion	Acemannan	Stimulates hair follicle growth	(80)
Anti-aging effects	Aloe-emodin	Inhibition of collagen degradation	(81)
Analgesic effects	Acemannan	Modulation of pain pathways	(79)
Antifungal activity	Aloe-emodin	Inhibition of fungal growth	(82)
Anti-acne effects	*Aloe vera* gel	Reduction of sebum production and inflammation	(83)
Cardioprotective effects	Acemannan	Improvement of lipid profiles	(84)
Neuroprotective effects	Aloe-emodin	Protection against neuronal damage	(85)
Antihyperlipidemic effects	Acemannan	Reduction of cholesterol levels	(86)
Antidepressant effects	Aloe-emodin	Modulation of neurotransmitter levels	(87)
Antispasmodic effects	Acemannan	Relaxation of smooth muscle	(88)
Antihypertensive effects	Acemannan	Vasodilation and blood pressure regulation	(84)
Detoxification	*Aloe vera* gel	Enhancement of liver function	(89)
Anti-allergic effects	Acemannan	Inhibition of histamine release	(90)
Anti-psoriasis effects	*Aloe vera* gel	Reduction of skin cell proliferation	(47)

### 4.1 Anti-inflammatory and immunomodulatory effect

*Aloe vera* may exhibit a therapeutic effect in managing experimental colitis and cryptosporidiosis. In the TNBS-induced colitis model, *Aloe vera* reduced serum inflammation markers, including interleukin-6 (from 41 to 21 pg./mL), tumor necrosis factor-alpha (from 75 to 44 pg./mL), and nitric oxide (from 24 to 6 *μ*/mL) (40). Furthermore, the administration of *Aloe vera* gel (250 mg/day) resulted in a significant reduction of cryptosporidiosis in immunosuppressed mice, with complete eradication observed in 99 percent of the subjects. It was associated with a decrease in inflammatory cytokines, including IFN-gamma, IL-4, IL-6, and IL-17 (41). *Aloe vera* extracts have also been found to possess anti-inflammatory properties. The extracts of *Aloe vera* gel suppressed inflammatory mediators in rats subjected to a high-fat diet, including TNF-alpha, TGF-beta, and IL-6. *Aloe vera* peel nanovesicles have been shown to diminish the production of pro-inflammatory cytokines, including TNF-alpha, IL-1beta, and IL-6, as well as myofibroblast differentiation and collagen matrix contractility (42). The potential anti-inflammatory application of *Aloe vera* can be attributed to its bioactive components, which can diminish the inflammatory response. The results suggest that *Aloe vera* may serve as an effective treatment for inflammatory disorders. *Aloe vera* has been documented to affect immunological function in experimental models. Methanolic extracts decreased C-reactive protein levels and elevated total white blood cell counts in Wistar rats (43). Dietary supplementation in broilers has been linked to improved humoral responses to viral challenges (44). These data indicate possible immunomodulatory activity, associated with the plant’s phytochemical components. Nevertheless, most of the information derives from animal research with inconsistent dosages and formulations, complicating the extrapolation of effects to people. Rigorous clinical trials are essential prior to drawing any treatment findings.

### 4.2 Dermatological and wound healing

*Aloe vera* gel is commonly used in skincare formulations, such as lotions, sunscreens, and cosmetics. The anti-inflammatory, antibacterial, and moisturizing properties make it advantageous for treating wounds, burns, and skin irritation (45). *Aloe vera* is acknowledged for its role in stimulating hair growth through the improvement of blood circulation to the scalp. *Aloe vera* exhibits anti-aging properties through its capacity to hydrate the skin and diminish the appearance of pores and wrinkles. *Aloe vera* extract in gel formulation improved skin hydration, reduced pore size, and decreased wrinkles. *Aloe vera* extract gel exhibits rejuvenating properties. *Aloe vera* exhibits antibacterial properties that contribute to acne prevention by decreasing the growth of acne-related bacteria and alleviating the inflammatory response. *Aloe vera* functions as a potent moisturizer, alleviating skin inflammation and accelerating the healing process. *Aloe vera* demonstrates potential anti-photoaging properties in bio-cellulose-based products (46).

*Aloe vera* contains vital components essential for the biological process of wound healing, including a plethora of essential amino acids and inorganic electrolytes such as iron, potassium, magnesium, chromium, copper, sodium, calcium, and zinc, which are necessary for tissue repair and restoration of integrity. These factors are crucial for cellular repair, regeneration, and the enzymatic activities involved in tissue remodeling. *Aloe vera* enhances the immune system by promoting antibody production and initiating tissue repair through the provision of growth factors (47). Numerous studies show that *Aloe vera* accelerates wound healing. It reduces scarring by stimulating cell growth and deep tissue healing. *Aloe vera* strengthens wounds by boosting collagen formation and collagen fiber organization and composition by reinforcing collagen cross-links (48). *Aloe vera*’s 99% water content makes it hydrate and decreases skin fragility. Mucopolysaccharides, amino acids, and zinc also hydrate skin, strengthen the skin barrier, reduce erythema, and prevent skin ulcers. *Aloe vera* improves wound healing speed and quality (49). The gel contains auxins and gibberellins, which encourage cell development and tissue regeneration. For chronic wound prevention, *Aloe vera* modulates cytokine expression and wound healing inflammation. *Aloe vera*’s antibacterial and antifungal capabilities prevent secondary wound infections, speeding healing and minimizing risk. *Aloe vera* is becoming desirable as an ingredient in topical gels, dressings, and ointments used in clinical and home wound management due to its natural, multi-faceted wound therapeutic efficacy (50).

### 4.3 Gastrointestinal and digestive health

*Aloe vera* has demonstrated considerable efficacy in improving digestive health, attracting attention from both traditional and contemporary medical practices. *Aloe vera* is known for its high levels of bioactive compounds, which contribute to its laxative effects and ability to promote bowel movements, thereby relieving constipation and positively influencing overall digestive health. Research demonstrates that polysaccharides present in *Aloe vera* can improve gut mucosal integrity and affect gut microbiota, thereby supporting a healthy microbiome crucial for effective digestive functions (51). *Aloe vera*’s anti-inflammatory properties may improve gastrointestinal conditions such as irritable bowel syndrome (IBS) and inflammatory bowel disease (IBD). *Aloe vera* exhibits medicinal properties that may reduce oxidative stress in the gastrointestinal system, which is a contributing factor to the development of digestive disorders. *Aloe vera* is considered an effective natural supplement for digestive health, owing to its diverse mechanisms, and should be used in conjunction with other dietary and lifestyle changes (52). *Aloe vera*’s anti-inflammatory properties may improve gastrointestinal conditions such as irritable bowel syndrome (IBS) and inflammatory bowel disease (IBD). *Aloe vera* exhibits medicinal properties that may reduce oxidative stress in the gastrointestinal system, which is a contributing factor to the development of digestive disorders. *Aloe vera* is considered an effective natural supplement for digestive health, particularly when combined with other dietary and lifestyle changes (53).

Constipation is a common gastrointestinal disorder affecting approximately 60% of pregnant women. *Aloe vera* has been studied as a potential intervention for constipation. A pre-clinical study indicated that *Aloe vera* tea (0.2 g/20 g body weight) significantly increased defecation frequency and exhibited a rapid onset of laxative effect in pregnant mice. The laxative effects of *Aloe vera* are attributed to its ability to stimulate bowel movements and relieve constipation (54). Traditional Chinese medicine utilizes *Aloe vera* for its cooling properties and bitter taste to relieve constipation. *Aloe vera* has demonstrated efficacy in relieving constipation in various populations, including cancer patients receiving morphine, with *Aloe vera* syrup acting as a cost-effective and practical alternative to traditional laxatives. A polyherbal formulation containing *Aloe vera* significantly alleviated constipation symptoms, demonstrating an 84% reduction after 1 month and a 90% reduction after 3 months in a double-blind, placebo-controlled clinical trial (55).

### 4.4 Metabolic disorder

Diabetes mellitus is a chronic condition characterized by metabolic dysfunction, specifically indicated by abnormal insulin and glucose levels in the bloodstream. The potential benefits of *Aloe vera* in the management of diabetes have received heightened scientific scrutiny. Research indicates that *Aloe vera* effectively reduces blood sugar levels by enhancing insulin sensitivity, improving glucose tolerance in peripheral tissues, and decreasing hepatic glucose production. Bioactive compounds in *Aloe vera*, including anthraquinones, are believed to improve glucose and insulin metabolism. *Aloe vera* may influence gut microbiota composition, potentially alleviating symptoms of type 2 diabetes (56) Evidence from multiple animal studies supports the anti-diabetic efficacy of *Aloe vera*. The extraction methods of methanol solutions utilizing *Aloe vera* flowers significantly reduced glucose levels in alloxan-induced diabetic rats, without causing hyperglycaemia or pathological weight gain (57). The ethyl acetate extract and its active components have been shown to increase insulin levels, enhance antioxidant defenses, and improve metabolic profiles in diabetic animals (58). A distinct study demonstrated that *Aloe vera* supplementation led to reductions in fasting blood glucose, triglyceride levels, and DPP-IV enzyme activity, while also increasing insulin levels in genetically obese rats (56). The anti-diabetic effects of *Aloe vera* are primarily associated with its antioxidant and anti-inflammatory properties, which reduce oxidative stress and chronic inflammation commonly experienced by individuals with diabetes. Current research indicates that *Aloe vera* may be a beneficial adjunct to conventional diabetes management therapies (59).

*Aloe vera* gel has been shown to reduce hyperlipidaemia, especially in those who did not react to diet alone. Clinical trials showed that *Aloe vera* gel treatment reduced total cholesterol by 15.4%, triglycerides by 25.2%, and LDL cholesterol by 18.9%. These findings suggest that *Aloe vera* may help treat dyslipidaemia and metabolic diseases (92). Studies on hypercholesterolemic rats showed that *Aloe vera* gel mixed with Lactobacillus rhamnoses was more effective. By limiting cholesterol production and intake, this combination improved lipid profiles. Gel-probiotic combination improved digestive function and reduced cardiovascular risk by lowering blood cholesterol (93). Phytosterols, fiber, and antioxidants in *Aloe vera* may prevent cholesterol absorption and stimulate its excretion, reducing lipids. Phytotherapy using probiotic-based products prevents cardiovascular illnesses and offers a novel treatment option for high-risk groups where traditional treatments fail (94).

### 4.5 Antifungal properties

*Aloe vera* demonstrates considerable antifungal efficacy and may be employed against several fungal diseases. Processed *Aloe vera* gel has been shown to effectively suppress the growth of *Candida albicans*, exhibiting potent anti-candidal activity attributed to a specific 14-kDa protein present in the gel. Moreover, the pulp of *Aloe vera* has demonstrated antifungal properties against specific plant diseases, including Rhizoctonia solani, Fusarium oxysporum, and Colletotrichum coccodes, mostly by inhibiting mycelial growth and development (60). Recent research has shown the extensive antifungal properties of bioactive phytochemicals in *Aloe vera*, including aloine and anthraquinone derivatives. The results validate the potential utility of the *Aloe vera* plant as a natural antifungal agent in medical and agricultural domains (61).

### 4.6 Antiviral properties

Key antiviral compounds in *Aloe vera* comprise acemannan, aloe-emodin, lectin fractions, anthraquinone, and aloin. Acemannan is notably recognized for its ability to modulate the immune response, thereby enhancing the physiological functions of macrophages, monocytes, and T-cells, which contributes to its inhibitory effects on herpes simplex virus (HSV) infections in laboratory settings (62). Aloe-emodin has demonstrated efficacy against viruses such as varicella zoster and influenza, suggesting that *Aloe vera* extracts may be beneficial in the treatment of these viral illnesses. The lectin fractions obtained from *Aloe vera* gel directly affected the proliferation of cytomegalovirus (CMV) by inhibiting its viral protein synthesis, demonstrating a clear antiviral potential (45). Furthermore, the effects of anthraquinones and aloin are regarded as virucidal, particularly against enveloped viruses, potentially interacting synergistically to enhance immunity. Recent investigations have validated these findings; specifically, *Aloe vera* gel extracts displayed significant antiviral activity against HSV-1, with a 5% concentration showing the highest efficacy. Molecular docking analyses indicate that phytochemicals, including triterpenoids and folic acid present in *Aloe vera*, exhibit a significant binding affinity for HSV proteins, highlighting the therapeutic potential of these substances (63). Collectively, these investigations highlight the multifaceted antiviral activities of *Aloe vera*, which integrate immunological boosting with direct suppression of viral activity.

### 4.7 Teeth and gum protection

*Aloe vera* is widely employed in dentistry, especially for addressing a range of oral conditions. This approach is notably effective in reducing pain and facilitating healing after periodontal flap procedures. *Aloe vera* has been shown to alleviate gum diseases, including gingivitis and periodontitis, by reducing gum bleeding, irritation, and edema. Mouthwashes and toothpaste with *Aloe vera* have shown considerable effectiveness in reducing plaque buildup and gum inflammation (64). It exhibits antiviral properties, demonstrating efficacy in the treatment of infections induced by herpes simplex and herpes zoster viruses. *Aloe vera* gel is effective for alleviating discomfort on mucosal surfaces behind dentures, attributed to its antifungal and cooling properties. It effectively alleviates discomfort caused by oral ulcers, particularly in the corners. Studies indicate that *Aloe vera* gel suppresses the growth of *Candida albicans*, a common fungal strain found in the oral cavity (65). *Aloe vera*-based tooth gels, in contrast to traditional toothpastes, do not possess abrasive properties, rendering them appropriate for individuals with sensitive teeth or bleeding gums. Additionally, acemannan, a complex polymer obtained from mannose-rich powder of the *Aloe vera* plant, exhibits natural viscosity and is recommended as an effective denture adhesive (66). Research indicates that its adhesive properties are significantly high while exhibiting minimal cytotoxicity. Topical *Aloe vera* gel has been shown to effectively treat individuals with lichen planus, a chronic inflammatory condition of the oral cavity (67).

### 4.8 Safety and toxicological considerations

*Aloe vera* is considered safe, especially when applied topically using the inner leaf gel or consumed in moderate oral doses. Acute and subacute toxicity studies conducted on mice and rats indicate a high level of tolerability, as evidenced by the absence of mortality, behavioral changes, or significant modifications in hematological, biochemical, or histopathological parameters, even at elevated doses of up to 15 g/kg body weight (68). Genotoxicity assessments, such as Ames tests, micronucleus assays, and sperm morphology analyses, have consistently demonstrated no evidence of mutagenic or genotoxic effects for *Aloe vera* preparations at the concentrations tested (9). The findings indicate that *Aloe vera* is relatively safe as a dietary supplement and functional food ingredient when consumed within controlled dosages.

Certain components of *Aloe vera*, especially the anthraquinone-rich latex, present potential risks. The prolonged or excessive intake of anthraquinones, including aloin and aloe-emodin, is linked to gastrointestinal disturbances from laxative use, as well as hepatotoxicity, nephrotoxicity, phototoxicity, and reproductive toxicity, which encompasses teratogenic effects during pregnancy (61, 69). Topical application is generally well tolerated; however, rare instances of burning, stinging, redness, or generalized dermatitis have been documented, frequently associated with anthraquinone content. The safety profile is contingent upon preparation and dosage: gel-based products are typically safe, while whole-leaf extracts or high-dose supplements necessitate caution (70). *Aloe vera* exhibits a favorable safety profile in preclinical studies. However, it is crucial to consider dosage, formulation, and population-specific risks, such as those associated with pregnancy and chronic use, for its therapeutic and dietary applications.

## 5 Conclusion

The therapeutic properties of *Aloe vera* support its use in traditional and alternative medicine. The research supporting its effectiveness in oral health, especially in cavity prevention and chronic periodontitis management, underscores its versatility and accessibility as a natural remedy. The nutritional assessment of *Aloe vera* indicates its beneficial components that could enhance health outcomes, highlighting its potential in tackling various health concerns. *Aloe vera* offers a cost-effective solution with minimal side effects for improving patients’ overall health. The increasing number of studies supporting its use reinforces the plant’s status as a significant natural resource and prompts healthcare practitioners to consider it as a viable therapeutic option.
